# CUSTODIAL KNOWLEDGE AND LEGAL PERCEPTION AMONG GRANDPARENTS RAISING THEIR GRANDCHILDREN

**DOI:** 10.1093/geroni/igz038.143

**Published:** 2019-11-08

**Authors:** Elizabeth Bownes, Martha R Crowther, Jennifer Cox

**Affiliations:** University of Alabama, Tuscaloosa, Alabama, United States

## Abstract

The current study examines the ways in which grandparents raising their grandchildren (GRCs) understand custody, perceive the legal system, and access resources related to their grandchild(ren)’s welfare. Due to the detrimental impact of the opioid crisis over the last decade, the number of skipped generation households is growing significantly not only in Alabama, but across the U.S.. Many GRCs lack crucial information regarding custody arrangements in a general sense or as it applies to education, healthcare, mental health, and financial aid. Critical gaps remain present in the GRC literature necessary to aid in future intervention studies and promote more effective support, resources, and policy for this population. The present study sought to examine the unique needs and experiences of GRCs, and to specifically explore legal aspects associated to their grandchild(ren)’s welfare. Using a mixed methods approach, GRCs in Tuscaloosa, Alabama completed a quiz of custodial knowledge, a survey on legal perception, and a semi-structured interview. Quantitative data revealed the most and least commonly understood aspects of custody, as well as opinions on associated legal systems related to a child’s “best interest.” Qualitative data analysis revealed the common themes related to custody to be unexpected assistance, ineffective assistance, leniency for parental deviance, fear of losing custody, and time and cost demands.

